# Supplementary material for: Cross-context qualitative synthesis of a mobile obstetric referral emergency system (MORES) in Ghana and Liberia
Source: PLoS One. 2026 Jun 4;21(6):e0350823. doi: 10.1371/journal.pone.0350823 (PMC13235915; doi:10.1371/journal.pone.0350823)
Supplement: S2 File — (DOCX) [file pone.0350823.s002.docx]

**Liberia**

**Individual Interview Guide**

**Facility Name**:  **Participant ID Number**:

**Date**: **Role**:

**Age**: **Professional Service**:

Now I would like to ask you a few questions about the WhatsApp program you have been using.

1. In your own words, tell me the purpose of this WhatsApp program.
2. How well do you think you understand the WhatsApp program?
3. How helpful/useful do you think the application is for you?
4. How helpful/useful do you think the application is for caring for pregnant women?
5. How often do you think you would use the WhatsApp platform?
6. What is good about the program?
7. What is difficult about using the program?
8. What could make the program better?
9. How much support do you need to use the program?
10. How was WhatsApp helpful to prepare for a referral?
11. How does the program support communication between your clinic and the referral hospital?
12. Was feedback from the referral hospital helpful? (only for rural health facilities workers)
13. What was the average time it took you to get feedback from the referral facility? (only for rural health facilities workers)
14. Other comments?

**Ghana**

**Focus Group Semi-Structured Discussion Guide**

1.What is the referral system in this district like?

**Probe**: Please describe the process, step by step, from the rural facility to this place

2. In our own words, what is the purpose of the WhatsApp program?

3. How well do you understand the WhatsApp program?

**Probe:** Please describe the process of the referral, from the rural facility to the district level.

4. How helpful has the WhatsApp program been for you?

**Probe:** Could you give an example of how the WhatsApp program was helpful?

5. How helpful or useful do you think the WhatsApp application has been in caring for pregnant women?

6. What is good about the WhatsApp application?

7. What is the bad side about the WhatsApp system?

8. How often do you think you would use the WhatsApp application?

9. What challenges do you having in using the WhatsApp application?

**Probe:** Can you give an example?

10. How do you think we can make the WhatsApp messaging system better?

11. What support do you need to use WhatsApp?

12. Would you recommend the WhatsApp system to other facilities?
