# Supplementary material for: Cross-context qualitative synthesis of a mobile obstetric referral emergency system (MORES) in Ghana and Liberia
Source: PLoS One. 2026 Jun 4;21(6):e0350823. doi: 10.1371/journal.pone.0350823 (PMC13235915; doi:10.1371/journal.pone.0350823)
Supplement: Attachment — Submitted filename: Response to Reviewers.docx [file pone.0350823.s004.docx]

Response to Reviewers

| **Editor Comments** | **Response to Editor** |
| --- | --- |
| The reviewers have raised a number of major concerns. They feel the manuscript should clearly justify the substantial difference in sample size and data collection methods. | Thank you for your response. We clarified that our study is a “cross-context synthesis” rather than a comparative analysis due to the difference in sample size (title, abstract line 19, and strengths and limitations 716). We believe this will help temper overinterpretation of the findings. |
| **Reviewer #1 comments** | **Response to reviewer #1** |
| While the Methods section later clarifies that this study is a secondary analysis of previously collected qualitative data, the abstract reads as though primary data collection was conducted specifically for the current study (e.g., “interviews were conducted,” “midwives participated in a focus group”). Given the methodological implications of secondary qualitative analysis, this distinction should be made explicit in the abstract for transparency and accurate interpretation | Thank you for pointing out our lack of clarity about the nature of the study in the abstract. The abstract on page 1-2 has been corrected to clearly reflect the study design.  Line 14-17 now reads, “To address the delayed provision of obstetric emergency care in Ghana and Liberia, our team previously implemented a mobile obstetric emergency referral system (MORES) connecting rural health facilities (RHFs) and district hospitals through a South-South-North triangular collaboration. This secondary qualitative data analysis aimed to synthesize the perceived barriers and benefits associated with the adoption of MORES in Ghana and Liberia.” |
| The manuscript is framed as a comparative analysis; however, the datasets differ substantially between countries. Liberia’s data comprise 62 individual interviews across both rural health facilities and district hospitals, while Ghana’s data derive from a single focus group discussion with 11 midwives at one district hospital. These differences limit the extent to which findings can be directly compared across countries. The authors are encouraged to more clearly justify the comparative framing, temper comparative claims, or reframe the analysis as a cross-context qualitative synthesis. | Thank you for your thoughtful feedback regarding the framing of the paper. We have adjusted the wording to frame the paper as a cross-context synthesis.  The title was changed to: **“Cross-context qualitative synthesis of a mobile obstetric referral emergency system (MORES) in Ghana and Liberia”**  Line 19 reads, “To guide this cross-context synthesis, we utilized Rogers’ Diffusion of Innovation Theory.”  Further in the **Strengths and Limitations** section, a distinction is made to list the different data sources as a limitation.  Line 715-716 now reads, “Therefore, the findings of the study should be interpreted as a cross-context synthesis rather than a comparison across countries.” |
| The manuscript reports frequency counts and percentages for Liberia while using raw reference counts for Ghana. Given the substantial difference in sample size and data collection methods, numerical comparisons across countries should be avoided or more clearly contextualized to prevent overinterpretation. | We agree it is important to contextualize our methods to prevent overinterpretation. We believe the frequency counts, percentages and reference counts increase transparency in our conclusions since the data set sample sizes significantly differed between the countries. The difference in sample size in addressed in our **Strengths and Limitations** section.  We added more explanation as to why the sample sizes were different in the **Methods Line** 312-313 **“**For efficiency, data were collected via focus group discussion in Ghana versus individual interviews in Liberia.” |
| The rationale for restricting public access to transcripts due to ethical considerations is understandable; however, the Data Availability Statement would benefit from clearer alignment with PLOS ONE’s policy language. Clarifying whether de-identified excerpts or controlled access might be possible would strengthen transparency. | Thank you for your comment. We have revised our data availability statement to further clarify.  “The research data is not available online due to the wording in the participant consent. Upon reasonable request, the data may be available. Please email the corresponding author and the open access data repository at the University of Michigan ([deepblue@umich.edu](mailto:deepblue@umich.edu)) with such requests.” |
| Some global statistics in the introduction (e.g., smartphone subscriptions) should be carefully checked for accuracy. It probably should be 7.4 billion and not million as stated. | Thank you for catching this typo. We updated this statistic further.  Line 54-55: “By 2028, there will be a projected 7.9 billion smartphone mobile network subscriptions worldwide.” |
| **Reviewer #2 comments** | **Response to reviewer #2** |
| The authors should avoid doing citations in the Methodology section and never in the Conclusions section. | Thank you for your comment. The citation was removed from the conclusion section. We feel the citations in the methodology section establishes credibility. Since this is a secondary analysis, we acknowledge the previous work our team has done through citations. |
| The Results seem to point that the Diffusion of Innovation Theory may not be the most appropriate theoretical framework as three of its categories are not significantly touched on and that there are themes captured but are not in the theory. Perhaps this should be explained better in the Discussion section rather than just mentioned as a matter of fact. | Thank you for your insightful comment. Through inductive analysis of the codes in the transcripts, the three constructs complexity, trialability, and observability were not discussed. We cannot be certain that those constructs did not influence adoption, only that they were not brought up in the interviews. Roger’s Diffusion of Innovation was extremely helpful for our understanding of the ways constructs hinder or encourage adoption. We have added further clarity regarding your point.  Lines 560-564 now reads: “In the context of MORES, the compatibility of WhatsApp and the relative advantage of utilizing MORES were prominent themes. However, Roger’s constructs of complexity, trialability, and observability were not themes that emerged inductively from the transcripts. Rogers highlights that while all five qualities of an innovation are meaningful for adoption, relative advantages and compatibility are of paramount importance [29].” |
| The differences in methods of data collection between Liberia and Ghana are mentioned as part of the study limitations but how such differences impact the findings need to be further elaborated. | Thank you for this comment. Reviewer 1 also highlighted this point and we agree a reframing of the study to avoid overinterpretation is necessary. We changed the language around this study being a comparison to being a “a cross-context synthesis” (title, abstract line 19, and strengths and limitations 716). |
| Still in the Strengths and Limitations Subsection, the two paragraphs on limitations seem to be contradicting with each other. The first paragraph says that the results should be interpreted carefully yet the second paragraph is saying that the findings can be generalized. These should be reviewed. | We appreciate you highlighting our contradiction. The language in the **Strengths and Limitations** section has been edited for clarity.  Lines 642-655, end of paragraph 1 and paragraph 2 in this section read: “There was unequal representation from each country. In Liberia, 62 healthcare workers were interviewed and 11 healthcare workers participated in the focus group Ghana. Therefore, the findings of the study should be interpreted as a cross-context synthesis rather than a comparison across countries.  While the qualitative data differed in structure from each country, a strength of the study is the inclusion of healthcare workers’ perspectives with similar systems in Ghana and Liberia. Ghana and Liberia both employ a referral process between basic emergency obstetric and newborn care (BEmONC) facilities and comprehensive emergency obstetric and newborn care facilities (CEmONC) when an escalation of care is needed for blood transfusion and or cesarean section [19,47,52]. A notable strength of this study is the applied cross-country learnings.” |
